# Written standard sentence materials convey social information

**DOI:** 10.1121/10.0007466

**Published:** 2021-12-21

**Authors:** Alayo Tripp, Benjamin Munson

**Affiliations:** Department of Speech-Language-Hearing Sciences, University of Minnesota-Twin Cities, Minneapolis, Minnesota 55455, USA tripp158@umn.edu, munso005@umn.edu

## Abstract

The Harvard/IEEE (henceforth H/I) sentences are widely used for testing speech recognition in English. This study examined whether two talker characteristics, race and gender, are conveyed by 80 of the H/I sentences in their written form, and by a comparison set of sentences from the internet message board Reddit, which were expected to convey social information. As predicted, a significant proportion of raters reported perceiving race and gender information in the H/I sentences. Suggestions of how to manage the potential influence of this social information on measures of speech intelligibility are provided.

## 1. Introduction

Speech intelligibility is a widely used summary measure of speech understanding. Speech intelligibility is affected by myriad factors. Critical to the current investigation is the finding that it matters what *materials* you use to test speech recognition. In a classic study, Miller *et al.* (1951) showed that sentences are more intelligible when listeners can use context to predict upcoming words. Given that listeners may treat the identity of the person who produced some speech as context for predicting their speech, it becomes necessary to examine whether individuals' beliefs about the person who produced a sentence might influence measures of speech intelligibility. Consistent with this, the modern study of speech perception has found that *person* perception and *speech* perception are intertwined (Babel and Russell, 2015; McGowan, 2015): the intelligibility of the same set of speech materials varies depending on the type of person to which individuals are listening. We use the term *person perception* over the more-conventional *talker perception* because person perception is not modality-limited and acknowledges that personae are perceived through other language modalities, like sign and writing.

The Harvard/IEEE (henceforth H/I) sentences are a widely used source of speech material used for testing speech recognition in English, and they have been adapted into other languages (Aubanel *et al.*, 2014; Aubanel *et al.*, 2020). The H/I sentences were developed to test communications technology and to determine the effects of distortion and noise on intelligibility (Institute of Electrical and Electronics Engineers, 1969). They were later adapted for assessment of speech perception in basic science and clinical research studies. They were published as part of the *Recommended Practices for Speech Quality Measurement*, as part of an effort to facilitate the comparison of data obtained in different laboratories. While the original 1969 report indicates that its recommendations are “[i]ntended … not as a long term standard,” (p. 1), they nonetheless have become ubiquitous in audiometric work, including research and development of assistive technologies, like hearing aids and cochlear implants for deaf and hard of hearing (D/HoH) persons (Zhang, 2015).

The H/I sentences are divided into sets of 10 sentences. Each sentence set is designed to be homogenous in structure, and the different lists are meant to be psychometrically equivalent. Each set contains 10 syntactically well-formed sentences containing common words which are expected to be familiar to speakers proficient in English. Each sentence contains five keywords: a single, two- syllable word, and four monosyllabic words. The sentences are constructed to have low probability, i.e., containing relatively uncommon sequences of words. Sentences with relatively lower probability are of higher difficulty for an individual to predict or repair using context; therefore, providing a better measurement of the individual's ability to directly recognize presented speech forms. The sentences were constructed to be *phonetically balanced*, reflecting the frequency with which speech sounds appear in the language in word-initial, word-medial, and word-final positions. In order to achieve the best possible measurement of speech intelligibility, it was desired that the test materials represent the true distribution of sounds in the English language as closely as possible.

Social factors were not explicitly considered in the design of the H/I sentences. That is, the sentences were not designed to be equally likely to have been uttered by different groups of people. In light of this design choice, it is possible that individuals may perceive the content of these sentences to imply social characteristics of the people who produced them, such as age, gender, race, and ethnicity. Given the demographics of academia and industry, especially at the time that these materials were constructed, we predicted that the sentence content in their written form would be most likely to suggest the authorship of White^1^ men.

Why might it matter if standard sentence materials convey the authorship of white men? Studies of speech perception have long found that measures of speech intelligibility may be affected by social differences between speakers of different races and genders. Studies have claimed that white people produce the most intelligible speech, and education improves the chance that a speaker will be deemed intelligible by listeners outside their race (Eisenberg *et al.*, 1968). White listeners have also been shown to have less ability to successfully interpret Black speech than vice versa (Harris Nobber and Seymour, 1979; Babel and Russell, 2015) found that the speech of native speakers of mainstream Canadian English was more intelligible to listeners when it was paired with static pictures of their faces only if they were white. When speech was paired with pictures of ethnically Chinese talkers, speech became less intelligible, perhaps because listeners had racialized expectations of the variety of language people would use. Similar findings are reported by McGowan (2015) on the influence of visual primes on the perception of Chinese-accented English. Gender also influences intelligibility. While many of these effects appear to be due to the effect of sexual dimorphism on the acoustic characteristics of cisgender men and women's speech, there is evidence that gender differences in intelligibility persist when these acoustic variables are controlled statistically (McCloy *et al.*, 2015), which may reflect the effect of social evaluations of gender on intelligibility. Van Berkum *et al.* (2008) present neurophysiological evidence that listeners identify social characteristics of voices rapidly, and that these guide their expectation of what the person should say. This leads to conflicts when the voice and the message are not consistent with the same social characteristics.

Studies like Babel and Russell (2015), McGowan (2015), McCloy *et al.* (2015), and Van Berkum *et al.* (2008) inspire us to ask how perception of a spoken sentence might proceed if the content of the sentence conveys the race and gender of the author. The possibility that sentence content implies race and gender is well grounded in previous literature. Participants make judgments of ethnicity and gender of imagined speakers, and differences in these judgments can be predicted on the basis of the participant's social identity (Merritt and Harrison, 2006). Even imagined gender can alter phonetic perception of a disembodied voice (Johnson *et al.*, 1999). The current study contributes to this literature by examining whether two talker characteristics, race and gender, are suggested by the H/I sentences in their written form. We also included a comparison set of sentences from an internet message board, Reddit. We included the Reddit sentences because we reasoned that because they were generated by users in the course of discussion conveying their personal experiences and opinions, they would represent a potentially more naturalistic set of English sentences and would be more likely to convey race and gender. By including the Reddit sentences as an intentionally social “anchor”, we could be more confident that a finding that the H/I sentences imply race and gender did not occur simply because participants did not have an “anchor” of socially imbued sentences against which to compare them. We also collected a measure of the naturalness of the sentences, to examine whether any sentences judged to suggest race/ethnicity or gender would also be perceived as especially natural or unnatural.

## 2. Methods

### 2.1 Sentence materials

The specific H/I sentences included 80 items. These were assembled by taking eight of the 10-item lists and replacing a small number of sentences whose content was potentially deeply objectionable (i.e., *The clan gathered on each dull night*), which could be interpreted as referencing the Ku Klux Klan).

Reddit is a large online forum which hosts numerous distinct communities, or *subreddits*, whose individual titles are conventionally described using the label r/[topic].We collected sentences from numerous subreddits explicitly associated with topics of race, ethnicity, and gender, including r/AsianAmericans, r/BlackLadies, r/ABCDesis, and r/mixedrace. We also selected several general interest subreddits, e.g., r/technology, r/science, and r/worldnews. This selection of subreddits was chosen to ensure that our sample of sentences would reflect a diverse demographic distribution of Reddit users. Of the aggregated sentences, a subset of 80 was chosen by quasi-random selection. It included sentences of the same general length of the H/I sentences, and excluded sentences written in the first or second person, and sentences judged by consensus to be confusing or inflammatory in content. These sentences were also corrected for typos so that their presentation would match the H/I sentences as closely as possible. See the supplementary material for the full set of sentence materials for this study.^2^

### 2.2 Participants

Recruitment for the experiment was conducted through the website, *Prolific Academic*. Individuals were invited to participate if they were 18–70y , spoke English as their first language, and were in either the United States or Canada. A total of 162 individuals participated. The mean age was 32.9 y (SD = 12.2). The gender of the 162 individuals was 82 male, 78 female, 1 nonbinary, and 1 genderqueer. Individuals reported their race and ethnicity using the two questions from the U.S census. The racial breakdown was 108 White, 17 Black, 11 East Asian, 3 South Asian, 1 Other Asian, 2 Indigenous, 18 More than one Race, and 1 chose not to report race. The ethnicity breakdown was 19 Latinx (8 White, 2 Indigenous, 8 More than one Race, 1 chose not to report race) and 143 non-Latinx.

### 2.3 Methods

The experiment was programmed in Qualtrics (www.qualtrics.com). The introduction to the entire experiment explained that the broad goal of the authors' [Tripp and Munson (2021)] research was to understand how audiovisual speech intelligibility is affected by talker race, and that we were concerned about the possible confounding factor of information conveyed by the sentence. The procedures in this study were determined by the University of Minnesota Institutional Review Board not to be human subjects research, as defined by US law. This determination was disclosed to participants in the study instructions, which included key elements of the informed consent process (the purpose of the study, the use of the data, expected duration, procedures to be used, and the voluntary nature of the task) as defined in section 116 of U.S. law 45 CFR 46. An especially relevant passage in the instructions is below:

“Because [our future studies will present] our sentences audiovisually, and because our talkers will be diverse in race, ethnicity, and gender, we have to consider something that people haven't considered before: whether specific sentences convey anything about the speakers who produce them. That is, imagine a sentence like ‘I'm so excited that my husband and I just bought a million-dollar home in rural Vermont.’ If you read that sentence, you might assume things about the gender of the speaker (since there are many more women married to men than men married to men), and perhaps something about the race or ethnicity of the speaker (since Vermont has the second-highest percentage of White residents of the 50 U.S. states). We would like to pick a set of sentences that are equally likely to be produced by a person regardless of race or gender. We also want to pick sentences that are plausible. To accomplish this, we are conducting this exercise, in which you will read sentences and make some ratings about how surprised you would be to hear the sentence in the current calendar year, and whether the sentence conveys anything about the race and gender of the person who produced it. These ratings will help us pick the right sentences for our research.”

On each trial, a sentence was presented and participants were asked three questions. First, they were asked about its naturalness using the question, “*How surprised would you be to hear this sentence produced by someone in the current calendar year?*” The responses were, “*not at all surprised*,” “*somewhat surprised*,” “*very surprised*,” and “*extremely surprised.*” This question was selected in order to target judgement of naturalness in sentences over judgements, with the expectation that familiarity with specific language may influence these judgments. Rather than focusing on how novel the presented sentence may be in form or in content, we instead requested participants to provide holistic impressions regarding the congruency of the speech with utterances that they hear in their everyday lives. We anticipated that asking broadly about the quality of the sentence as surprising would be more easily and consistently understood, and more quickly answered than direct inquiries about naturalness or frequency.

Participants were also asked. “*If you had to guess, would you be fairly certain of the race or ethnicity of the person who produced this sentence?”* If they clicked “yes”, a menu appeared, asking them to indicate a selection from the following: *White (non Latino/a)*, *Latino/a of any race*, *African American/Black*, *South Asian (Afghanistan, Bangladesh, Bhutan, India, Maldives, Nepal, Pakistan, or Sri Lanka)*, *East Asian (Mongolia, China, Japan, or Korea)*, *Native American/Native Alaskan*, *Pacific Islander* and *Other*. The choice of race/ethnicity categories was motivated by the design of a parallel study on the effects of race and ethnicity on audiovisual speech intelligibility. The categories in this question were different from those used in the U.S. census, which the participants used to describe themselves.

They were also asked, “*If you had to guess, would you be fairly certain of the gender of the person who produced this sentence?* If they said “yes”, they were given the opportunity to select from three genders: *male*, *female*, and *a gender which is neither male nor female.* The order of the sentences was fully randomized. See the supplementary material for the entire set of Qualtrics materials for this study.^2^

## 3. Results

### 3.1 Naturalness

Responses to the question about naturalness differed considerably across the two sentence types. As expected, the Reddit sentences were perceived to be relatively natural: 77.7% of responses were *“not at all surprised*,” 14.8% “*somewhat surprised*,” 5.5% “*very surprised*,” and 2.1% “*extremely surprised.”* For the H/I sentences, 51.4% were *“not at all surprised*,” 28.2% “*somewhat surprised*,” 0.9% “*very surprised*,” and 6.5% “*extremely surprised.”* This difference was significant in a χ^2^ test of independence, χ^2^_[df=3]_ = 1999.8, p <0.001.

The difference between sentence types were remarkably consistent across the 162 participants. Only nine individuals provided more “*not at all surprised*” responses (indicating greater perceived naturalness) to the H/I sentences than to the Reddit sentences, and those individuals provided relatively similar responses overall to the two lists. In contrast, 56 individuals provided two or more times as many ratings in this category to the Reddit sentences than to the H/I sentences. Only three of the 162 participants judged all 160 sentences to be fully natural (i.e., giving them all ratings of *“not at all surprised*”). At the item level, 17.5% of the H/I sentences received 10% or more ratings of *extremely surprised*, indicating great unnaturalness. Only 2.5% of the Reddit sentences received 10% or more ratings for this category, and 12.5% of the Reddit sentences received no ratings in this category.

### 3.2 Race/Ethnicity

Similar proportions of the Reddit and H/I sentences were judged to indicate information about race and ethnicity. For the question, “*If you had to guess, would you be fairly certain of the race or ethnicity of the person who produced this sentence*?“, 17.3% of the responses indicated that people would be fairly certain of the race or ethnicity of the person who produced the H/I sentences, and 18.2% of the raters for the Reddit sentences. A logit mixed-effects model [fit using the lme4 package in R (Bates *et al.*, 2015)], with significance tests computed using the package LmerTest ( Kuznetsova *et al.*, 2017), with Satterthwaite's approximation for degrees of freedom] examined whether sentence type (contrast-coded, H/I = −1; Reddit = 1) affected responses to the race/ethnicity question. The model included random intercepts for sentence and participant, and a random slope (uncorrelated with intercept) for the effect of sentence type on participant. This model fit the data better than a baseline model with only random intercepts for sentence and participant (χ^2^_[df=2]_ = 71.21; p < 0.001); however, the coefficient for sentence type was not significant in the full model (β = 0.053; SEM = 0.060; z = 0.89; p = 0.38). Hence, the improved fit of the full model was because of the random effect of sentence type on rater. Consistent with this, 21 participants answered “no” to all 160 items, and an additional 13 participants gave only one “yes” response. Of the remaining 96 individuals, 73 individuals provided as many or more “yes” ratings to the Reddit sentences than to the H/I sentences and 56 gave more “yes” responses to IEEE sentences. This was as expected, given that the Reddit sentences were expected to contain social information. Critically, 42 of the participants provided 20% or more responses of “yes” to the H/I sentences.

At the item level, 13 H/I and six Reddit sentences received fewer than 10% “yes” responses; 23 of the H/I and 27 of the Reddit sentences received more than 20% “yes” responses. We used a χ^2^ test of independence to examine whether the specific race responses (including both the eight categories listed in the question for a “yes” responses, and a ninth category for the “no” responses) differed as a function of sentence type. This test was significant in a χ^2^ contingency test, χ^2^_[df=8]_ = 160.45, p < 0.001. This was also true when the ‘no’ responses were excluded, χ^2^_[df=7]_ = 157.21, p < 0.001. For both sentence types, the bulk of the race categories (excluding the ‘no’ responses) were “white” (H/I 68.7%, Reddit 69.7%). Seven of the H/I sentences were identified by 20% or more of the participants as indicating the person who produced it was White. Of the remaining categories, the biggest difference between sentence types was in the percentage of responses that indicated that the author was African American/Black (7.4% H/I, 13.4% Reddit).

The majority of the participants (62%, n = 100) in this experiment were White and non-Latinx. The predominance of judgments that the sentences suggest whiteness might have been driven by the performance of that subset of participants, if they were judging sentences based on their own likelihood of producing them. To examine this, we evaluated a logit mixed-effects model predicting a binary variable coding whether the sentence was identified as being produced by a White person (i.e., a 1 for answers of “yes” and subsequent judgments of “white”, and a 0 for all other responses). The fixed effect was a contrast-coded variable indicating whether or not the participant was white and non-Latinx. Participant and item were random effects, and there was a random slope for the effect of participant whiteness on sentence. In this model, the coefficient associated with participant whiteness was not significant (β = −0.084, SEM = 0.155, z = −0.54, p = 0.59). This was also true when only the H/I sentences were examined (β = −0.133, SEM = 0.146, z = −0.91, p = 0.36). Hence, the preponderance of white responses does not appear to reflect the preponderance of white participants.

Finally, we examined whether distributions of race ratings for the H/I sentences differed systematically as a function of the race of the rater. For this analysis, we binned the racers into five categories, based on their responses to the two U.S. census questions on race and ethnicity. Table 1 shows the distribution of ratings. A contingency test showed that the ratings differed significantly across the five categories of raters, χ^2^_[df=32]_ = 336.95; p < 0.001.

**Table 1.** Distribution of ratings of race/ethnicity (rows) by participant race/ethnicity (columns).

Rated race/ethnicity	White, non-Latinx (%)	Latinx of any race (%)	Black, non-Latinx (%)	Asian, non-Latinx (%)	More than one race, non-Latinx (%)
White, non-Latinx	11.5	17.6	13.5	6.0	11.6
Latinx of any race	0.9	1.8	0.2	0.2	0.3
African-American/Black	1.2	3.5	1.2	0.4	0.9
South Asian	0.8	0.8	0.5	0.4	0.2
East Asian	1.3	2.6	1.3	1.0	1.3
Native American	0.7	2.2	0.4	0.0	0.1
Pacific Islander	0.3	1.1	0.4	0.0	0.1
Other	0.2	0.0	0.0	0.0	0.0
Does not convey race	83.3	70.5	82.4	92.0	85.5

As this table shows, the distribution of responses does not suggest that different racial/ethnic groups were responding to the items with their own race as a comparison point. However, there are differences across the rater groups. Given the immense complexity of race as a socio-political construct, the interpretation of these differences is well outside of the scope of this paper, other than to say that they support Plaut's (2010) (see also Tripp & Munson, 2021) argument that a *diversity science* orientation is needed to understand differences in *beliefs about differences* of the sort elicited by this survey.

### 3.3 Gender

Similar proportions of the Reddit and H/I sentences were judged to indicate information about gender: For the question, “*If you had to guess, would you be fairly certain of the gender of the person who produced this sentence*?”, 29.0% of the responses indicated that people would be fairly certain of the gender of the person who produced the H/I sentences, and 24.8% of the raters for the Reddit sentences. A logit mixed-effects model examined whether sentence type (contrast-coded, H/I = minus –1; Reddit = 1) affected responses to the gender question. The model included random intercepts for sentence and participant, and a random slope (uncorrelated with intercept) for the effect of sentence type on participant. This model fit the data better than a baseline model with only random intercepts for sentence and participant (χ^2^_[df=2]_ = 66.678; p < 0.001); however, the coefficient for sentence type was not significant in the full model (β = −0.141, SEM = 0.084, z = −1.677, p = 0.09). Again, this suggests that the improved model fit was due to the effect of sentence type on individual participants. Seven participants gave no responses of “yes”, and an additional seven individuals gave only one “yes” response for the 160 sentences. Of the remaining 148 individuals, eight individuals provided the same number of “yes” responses to the Reddit sentences and the H/I sentences, and more than twice as many individuals provided as many or more “yes” ratings to the Reddit sentences than to the H/I sentences (N = 101) than the opposite (N = 40). This was not expected, given that the Reddit sentences were expected to contain social information and the H/I sentences were not expected to contain social information. Critically, 97 of the participants provided 20% or more responses of “yes” to the H/I sentences.

At the item level, only two H/I and two Reddit sentences received fewer than 10% “yes” responses; 49 of the H/I and 50 of the Reddit sentences received more than 20% “yes” responses. A χ^2^ test of independence to examine whether the distribution of responses across the three gender categories chosen by people who responded “yes” to the gender question differed as a function of sentence type. This test was not significant, χ^2^_[df=2]_ = 0.450, p = 0.80. For both sentence types, more responses of “male” (H/I 58.4%; Reddit 59.3%) were given than “female” (H/I 40.9%; Reddit 40.0%) or “a gender that is neither male nor female” (H/I 0.6%; Reddit 0.7%). Finally, we examined whether distributions of gender ratings for the H/I sentences differed systematically as a function of the gender of the rater (binning together the genderqueer and nonbinary raters). A contingency test showed that the gender ratings differed significantly across the three categories of rater gender, χ^2^_[df=4]_ = 38.99; p <0.001. The female raters gave a larger proportion of “male” ratings (59.7%) than the male (57.7%) or genderqueer/nonbinary (52.6%) raters. The genderqueer/nonbinary raters gave a larger proportion of “female” ratings (47.4%) than the female (38.6%) or male (42.2%) raters. The percentage of ratings of “a gender that is neither male nor female” was similarly low across the three groups (female: 1.6%; male: 0.1%; nonbinary/genderqueer: 0%).

### 3.4 Relations among measures

The last analysis examined relationships among responses to the questions about naturalness, race, and gender. For each pair of measures (race and gender ratings, gender and naturalness ratings, race and naturalness ratings), we calculated the contingency coefficient. The contingency coefficient is a continuous measure of the strength of the association between two categorical variables and is based on the χ^2^ statistic. We calculated these both for the two types of sentences together, and separately for the H/I and Reddit sentences.

When considering both types of sentences, all three χ^2^ tests were significant, indicating associations between the pairs of measures were associated at greater-than-chance levels. The contingency coefficient for the association between race and gender was higher (0.337) than those for the associations between gender and naturalness (0.122) or between race and naturalness (0.116). The same general pattern held when the two types of sentences were examined separately: the contingency coefficients for the association between race and gender were higher (H/I: 0.310; Reddit: 0.367) than the association between gender and naturalness (H/I: 0.120; Reddit: 0.106) and the association between race and naturalness (H/I: 0.148, Reddit: 0.103). The association between race and gender ratings is not surprising, given that these two features are co-constructed (Johnson *et al.*, 2012). The weak association of naturalness with race and gender shows that these social variables can be suggested by sentences of varying naturalness. That is, constructing low-likelihood sentences does not preclude the sentences conveying the race and gender of the author.

## 4. Summary and Discussion

Consistent with our hypothesis, we found that the H/I sentences conveyed two types of social information about their author: race/ethnicity and gender. While this was not true for all of the sentences or for all of the individuals providing the ratings, there was an appreciable proportion of both raters and items that conveyed this social information very clearly. We regard these findings as evidence that the social information implied by the sentences might be a significant source of variation necessary to characterize responses to the sentences in, for example, intelligibility tests in which these sentences are uttered by individuals whose gender and race/ethnicity might be discernible through their voices. The variation in perception of social information would be particularly problematic if there were a conflict between the information that is implied through the sentences and the information that is conveyed through the voice. Since the time that the H/I sentence materials were first developed, we have significantly advanced our understanding of sociophonetic knowledge, and there is growing evidence that person perception affects psycholinguistic processing, including speech intelligibility (e.g., Babel and Russell, 2015; McGowan, 2015). Based on those findings and the findings of the current study, we believe that conflicts between the information conveyed by voice and that suggested by sentences' content could impact the outcome and time course of speech perception and comprehension.

The rates with which the H/I sentences conveyed these pieces of information were similar to the rate with which a comparison set of sentences from the internet message board Reddit, which we hypothesized would be much more likely to convey the race and gender of their authors. We also estimated the naturalness of the sentences. As predicted, the Reddit sentences were perceived to be more natural than the H/I sentences. However, the extent to which participants rated items as conveying race/ethnicity and gender was only weakly related to their judgment of the sentence's naturalness. Together, these provide convincing information that the H/I sentences are not socially neutral, and that this lack of neutrality is not an artifact of the sentences' naturalness.

Whiteness and maleness were the primary race and gender terms reported for both types of sentences. This was true even for Reddit sentences that were taken from subreddits for non-white groups. For example, the sentence, “*Being outraged and edgy is the thing these days*” (talking about the television show *The Big Bang Theory*) was taken from the subreddit r/ABCDesi, which discusses issues about South Asia, many of the authors of which are South Asian. Nonetheless, of the people who said they were confident that they could guess the author's race/ethnicity, 97% said that the author would be white.

The data in this paper show that it is impossible to conclude that the H/I sentences can be treated as racially neutral stimuli. It is possible, however, that even in maximally ambiguous contexts, judgements of gender and race may be skewed, with either participants' individual biases, or their knowledge of societal biases predicting their attribution of social group membership. This phenomenon has been demonstrated previously (Merritt and Harrison, 2006). The notion that whiteness is a default is a central object of study in the emerging field of whiteness studies (Sue, 2006). The finding that the H/I sentences are perceived to suggest default whiteness is a testament to the extent to which they invoke the dominant culture.

There are at least three responses to the problems with the H/I sentences. One of these is to identify and use only those H/I sentences that are found not to convey gender or race/ethnicity. Table 2 provides specific examples of H/I sentences that represent extremes of naturalness and of conveying social information.

**Table 2.** Example H/I sentences exemplifying different perceived naturalness (i.e., low surprisal), gender, and race/ethnicity.

		Naturalness	
Attribute	Neutrality	High	Low
Gender	Neutral	Two blue fish swam in the tank.	Read verse out loud for pleasure.
	Not neutral	Her purse was full of useless trash.^a^	March the soldiers past the next hill.^b^
Race/Ethnicity	Neutral	It rained, snowed, and hailed the same morning.	These days, a chicken leg is a rare dish.
	Not neutral	Rice is often served in round bowls.^c^	A tame squirrel makes a nice pet.^d^

^a^ Of the people who said they could discern the author's gender, 57% said that it conveys “*Male”* and 43% said that it conveys “*Female.”*

^b^ Of the people who said they could discern the author's gender, 100% said that it conveys “*Male*.”

^c^ Of the people who said they could discern the author's race, 89% of people said this sentence conveys “*East Asian.”*

^d^ Of the people who said they could discern the author's race, 91% said that it conveys “*White.”*

As this table shows, it is possible to identify H/I sentences that the 162 participants in this study regarded as generally socially neutral, i.e., not implying the author's gender or race/ethnicity. Future endeavors could include extending this exercise to the entire set of H/I sentences, recruiting a more diverse group of participants to provide judgements, and allowing participants to report impressions of social information which they experience with less certainty. Such a project could attempt to define a subset of the H/I sentences which are appropriately socially neutral and which could be used with a wide range of talkers in speech perception studies. One potential problem with this solution is that the instructions for this study may have encouraged individuals to be conservative in their judgments, that is, to provide race/ethnicity and gender judgments only when they were highly confident. It may be that a different set of instructions would reveal that there are more H/I sentences that imply social attributes more subtly. Such an exercise could also include other sentence sets that were developed more recently, such as the Basic English Lexicon (BEL) sentences (Rimikis *et al.*, 2013). Currently, it remains unclear what role context effects, such as the presentation of stimuli in an experimental setting, or participants' own racial and gender identity might play in judgments of social neutrality. Further study is required to tease apart the mechanisms by which participants report perceiving social information in the H/I sentences. However, whether the social information is truly encoded in the sentence content, or if such perception is only induced through individual's contextualization of the stimuli (one that may relate systematically to specific aspects of that person's identity), we should reject the supposition that the H/I sentences are experienced as socially neutral.

A second response to the problems identified in this paper would be to say that they are not sufficiently problematic, especially in light of other aspects of their design that are seen as beneficial. One of these aspects is the H/I sentence's purported *phonemic balance*. The assertion that the H/I sentences are phonemically balanced is that the occurrence of phonemes in them matches measurements of phonemic frequency obtained by analyzing print media, along with three formal speeches given by former U.S. presidents (Dewey, 1923). However, modern techniques relying on analysis of spoken, rather than written language corpora, suggest that the phonemic balance of the H/I sentences is relatively poor when compared to spoken corpora (Aubanel *et al.*, 2020). Moreover, the notion of phonetic balance is based on a comparison to a normative sample, and that normative sample is based on a specific variety and, potentially, a particular genre. Defining what materials constitute valid examples of “the English language” is a political decision, and the specific materials which were consulted reflect ideological beliefs about what media and concomitant social identities rightly can be taken as representative of the language. Even these measurements ultimately serve to privilege dialects which conform to the standard language ideology. The linguist George Zipf wrote of Dewey's findings regarding the relative frequency of speech sounds:

“Yet even these cannot be said to represent any actually spoken language; nor was that the author's intent. Both the terms “English” and “Modern American” designate artificial languages which exist rather in spite of phonetic laws than because of them. Let us repeat again, that the present law of frequency can only be exactly demonstrated in an actually spoken dialect.” (Zipf, 1929).

Hence, the H/I sentences' claim to phonetic balance is flawed. Zipf's quote shows that researchers have known this for almost a century. In short, the assertion that the H/I sentences should be used because they demonstrate phonemic balance is not tenable.

A third solution to this problem is to begin the endeavor of constructing a new set of sentence materials that address the issues in this report prospectively. Such an endeavor would be able to address the historical failure to detail the selection of specific social groups and associated phonemic patterns in the construction of the H/I sentences, which reflects a hidden bias defining the selected groups as socially and linguistically normative.

Overall, the findings in this study demonstrate a fundamental finding of sociolinguistics: language varies across different communities of use. One sentence set cannot serve as a standard for all communities using a language. Finding a set of sentences that are appropriate for use in a broad population requires explicit tests of the sort outlined in this study and misses the generalization that real-world language use involves conveying identity alongside regular linguistic messages. Just as speech materials in one language cannot be used to test speakers of an entirely different language, speech materials that represent one specific population cannot be used to represent all populations. Our increased understanding of individuals' sensitivity to social cues signaling talker identity, and of how talker identity interacts with speech perception, requires us to question whether English language speech materials are universally valid tools, or ones that instead, must be validated for use with specific populations for specific purposes.

## Footnotes

^1^
This paper intentionally breaks with the conventions of the American Psychological Association and instead aligns with the recommendations of Diversity Style Guide (2021) by capitalizing Black, Latinx, and Indigenous but not white (The Diversity Style Guide).

^2^
See supplementary material at https://hdl.handle.net/11299/225199 for the full set of sentence materials for this study.

## References

[1] Aubanel, V. , Lecumberri, M. L. G. , and Cooke, M. (2014). “ The Harvard corpus: A phonemically-balanced Spanish sentence resource for audiology,” Int. J. Audiol. 53(9), 633–638. 10.3109/14992027.2014.907507 24863133

[2] Aubanel, V. , Bayard, C. , Strauß, A. , and Schwartz, J. L. (2020). “ The Fharvard corpus: A phonemically-balanced French sentence resource for audiology and intelligibility research,” Speech Commun. 124, 68–74. 10.1016/j.specom.2020.07.004

[3] Babel, M. , and Russell, J. (2015). “ Expectations and speech intelligibility,” J. Acoust. Soc. Am. 137, 2823–2833. 10.1121/1.4919317 25994710

[4] Bates, D. , Maechler, M. , Bolker, B. , and Walker, S. (2015). “ lme4: Linear mixed-effects models using Eigen and S4. [R package version 1.1–9],” https://CRAN.R-project.org/package=lme4 (Last viewed November 29, 2021).

[5] Dewey, G. (1923). *Relative Frequency of English Speech Sounds* ( Harvard University Press, Cambridge, MA), Vol. 4.

[6] https://www.diversitystyleguide.com (Last viewed November 29, 2021).

[7] Diversity Style Guide (2021). “ Diversity Style Guide,” https://www.diversitystyleguide.com/ (Last viewed November 29, 2021).

[8] Eisenberg, L. , Berlin, C. I. , Dill, A. , and Frank, S. (1968). “ Class and race effects on the intelligibility of monosyllables,” Child Dev. 39, 1077–1089. 10.2307/1127274 5704388

[9] Harris Nobber, E. , and Seymour, H. N. (1979). “ Speaker intelligibility of black and white school children for black and white adult listeners under varying listening conditions,” Lang. Speech 22, 237–242. 10.1177/002383097902200304

[10] Institute of Electrical and Electronics Engineers. (1969). “ IEEE recommended practice for speech quality measurements,” IEEE Trans. Audio Electroacoust. 17(3), 225–246. 10.1109/TAU.1969.1162058

[11] Johnson, K. , Strand, E. A. , and D'Imperio, M. (1999). “ Auditory–visual integration of talker gender in vowel perception,” J. Phon. 27, 359–384. 10.1006/jpho.1999.0100

[12] Johnson, K. L. , Freeman, J. B. , and Pauker, K. (2012). “ Race is gendered: How covarying phenotypes and stereotypes bias sex categorization,” J. Pers. Soc. Psychol. 102, 116–131. 10.1037/a0025335 21875229

[13] Kuznetsova, A. , Brockhoff, P. B. , and Christensen, R. H. B. (2017). “ lmerTest package: Tests in linear mixed effects models,” J. Stat. Softw. 82, 1–26. 10.18637/jss.v082.i13

[14] Merritt, R. D. , and Harrison, T. W. (2006). “ Gender and ethnicity attributions to a gender-and ethnicity-unspecified individual: Is there a people= white male bias?,” Sex Roles 54, 787–797. 10.1007/s11199-006-9046-7

[15] McCloy, D. R. , Wright, R. A. , and Souza, P. E. (2015). “ Talker versus dialect effects on speech intelligibility: A symmetrical study,” Lang. Speech 58, 371–386. 10.1177/0023830914559234 26529902 PMC4634571

[16] McGowan, K. B. (2015). “ Social expectation improves speech perception in noise,” Lang. Speech 58, 502–521. 10.1177/0023830914565191 27483742

[17] Miller, G. A. , Heise, G. A. , and Lichten, W. (1951). “ The intelligibility of speech as a function of the context of the test materials,” J. Exp. Psychol. 41, 329–335. 10.1037/h0062491 14861384

[18] Plaut, V. C. (2010). “ Diversity science: Why and how difference makes a difference,” Psychol. Inq. 21, 77–99. 10.1080/10478401003676501

[19] Rimikis, S. , Smiljanic, R. , and Calandruccio, L. (2013). “ Nonnative English speaker performance on the basic english lexicon (BEL) sentences,” J. Speech Lang. Hear. Res. 56, 792–804. 10.1044/1092-4388(2012/12-0178) 23275396

[20] Sue, D. W. (2006). “ The invisible whiteness of being: Whiteness, white supremacy, white privilege, and racism,” in *Addressing Racism: Facilitating Cultural Competence in Mental Health and Educational Settings*, edited by M. G. Constantine and D. W. Sue ( John Wiley & Sons, Inc, Hoboken, NJ), pp. 15–30.

[21] Tripp, A. , and Munson, B. (2021). “ Perceiving Gender While Perceiving Language: Integrating Psycholinguistics and Gender Theory,” in *Wiley Interdisciplinary Reviews* ( Cognitive Science, e1583) 10.1002/wcs.1583 34716654

[22] Van Berkum, J. J. , Van den Brink, D. , Tesink, C. M. , Kos, M. , and Hagoort, P. (2008). “ The neural integration of speaker and message,” J. Cogn. Neurosci. 20, 580–591. 10.1162/jocn.2008.20054 18052777

[23] Zipf, G. K. (1929). “ Relative frequency as a determinant of phonetic change,” Harvard Stud. Class. P. 40, 1–95. 10.2307/310585

[24] Zhang, S. (2015). “ The Harvard sentences secretly shaped the development of audio tech,” available at https://gizmodo.com/the-harvard-sentences-secretly-shaped-the-development-1689793568 (Last viewed March 29, 2021)

